# HIV Treatment in the Criminal Justice System: Critical Knowledge and Intervention Gaps

**DOI:** 10.1155/2011/680617

**Published:** 2011-07-12

**Authors:** Jaimie P. Meyer, Nadine E. Chen, Sandra A. Springer

**Affiliations:** AIDS Care Program, Infectious Diseases Section, Yale University School of Medicine, 135 College Street, Suite 323, New Haven, CT 06510-2283, USA

## Abstract

The criminal justice system bears a disproportionate burden of the HIV epidemic. Continuity of care is critical for HAART-based prevention of HIV-related morbidity and mortality. This paper describes four major challenges to successful management of HIV in the criminal justice system: relapse to substance use, homelessness, mental illness, and loss of medical and social benefits. Each of these areas constitutes a competing priority upon release that demands immediate attention and diverts time, energy, and valuable resources away from engagement in care and adherence to HAART. Numerous gaps exist in scientific knowledge about these issues and potential solutions. In illuminating these knowledge deficits, we present a contemporary research agenda for the management of HIV in correctional systems. Future empirical research should focus on these critical issues in HIV-infected prisoners and releasees while interventional research should incorporate evidence-based solutions into the criminal justice setting.

## 1. Introduction

The criminal justice setting provides vast opportunities for early diagnosis, prevention, and treatment of HIV [1, 2]. One in seven people living with HIV in the United States passes through the criminal justice system (CJS) each year [3], and incarceration is considered an independent risk factor for HIV infection [4]. For those living with HIV, history of incarceration is a strong predictor of nonadherence to HIV treatment and care [5, 6]. 

Correctional facilities thus bear a disproportionate burden of the HIV epidemic in the USA with a prevalence rate of HIV 3–5 times higher than surrounding communities [3, 7]. Though substantial need exists for management of HIV during incarceration, it is complicated by equally prevalent comorbid medical and psychiatric diseases, [8] potential lack of privacy around HIV testing and treatment, [9] inmates' frequent mistrust of the healthcare system, [10] and issues of control related to the prison environment itself [11]. Perhaps as a result, only an estimated one-third of HIV-infected inmates with a clinical indication for therapy receive combination antiretroviral therapy (HAART) during incarceration [12, 13].

Despite these obstacles, management of HIV in correctional settings has been shown to be feasible, acceptable, [10] and highly successful [7]. In Connecticut, an impressive 59% of HIV-infected inmates achieved an undetectable viral load (i.e., <400 copies/mL) by the end of their incarceration [14]. In this incarcerated cohort, clinical benefit was derived regardless of the type of HAART regimen prescribed [15]. Equal access to health care during incarceration also enables impoverished and minority subpopulations to overcome some healthcare disparities that exist outside prison walls [13, 16]. The highly structured nature of prison contributes to these successes, providing a relatively drug-free environment and granting opportunities to optimize medication adherence through directly administered antiretroviral therapy [17]. The provision of HAART in USA correctional facilities is also supported by a Supreme Court decision that established there cannot be “deliberate indifference” to the health needs of inmates [18] and by state laws that protect the rights to care of HIV-infected inmates in particular [19]. 

Unfortunately, benefits of prison-based HAART are rarely sustained upon return to communities [14, 20, 21]. In the Texas CJS, few prisoners filled prescriptions for their HIV medications [22] or enrolled in an HIV clinic within 30 days following release [23]. Upon release from prison, individuals face difficulties adhering to HAART and HIV-related care services because they are often distracted by basic subsistence needs and temptations of drug relapse [24]. Medication nonadherence is associated with serious negative individual and public health consequences. Although even intermittent HAART adherence may provide some immunologic or virologic benefit, [25] the Strategies for Management of Antiretroviral Therapy (SMART) study demonstrated that continuous therapy has superior long-term outcomes in terms of AIDS-related morbidity and mortality [26]. 

There is a paucity of data evaluating barriers to sustained antiretroviral therapy for HIV-infected persons transitioning from the CJS to the community. In this review, we outline major obstacles to continuous HIV care among populations who interface with the CJS, both during incarceration and after release. Major challenges include relapse to substance use, housing instability, comorbid mental illness, and coverage gaps in medical and social benefits. We briefly describe the significance of each of these issues in general CJS or in HIV-infected nonincarcerated populations and then explore potential areas for future empirical and interventional research. Rigorous scientific evaluation of these “knowledge gaps” is critical for progress in the field. Furthermore, success of programs designed to facilitate transition between prisons and communities hinges on bringing evidence-based solutions into the criminal justice setting.

## 2. Methods

A search strategy was undertaken using PubMed, OvidSP, and MEDLINE. The following key terms were used: *HIV, HAART, adherence, outcomes, prisoners, prison/jail, incarcerated, inmates, criminal justice, corrections, transition, linkage, and released.* These were combined with terms relevant to each topic of interest: *substance, addiction, drugs, alcohol, dependence/use/abuse, homelessness, housing, mental illness, depression, insurance, Medicaid, and benefits. *Continuity of care was defined in terms of HAART adherence and enrollment in HIV care upon release. Additional references from seminal papers were reviewed to broaden the search and assure that important contributions were not overlooked. Articles were included if they were written in English and were published in peer-reviewed journals between 1990–2011.

## 3. Results

### 3.1. Relapse to Substance Use

#### 3.1.1. Epidemiology of the Problem

Drug use and crime are inextricably linked. In the United States, where the “war on drugs” has devolved into a “war on drug users,” [27] drug-related offenses are punishable by incarceration. As a result, the USA, which contributes only 5% of the world population, holds 25% of the world prisoners [28]. In this context, almost half of USA prisoners screen positive for marijuana, cocaine, heroin, or methamphetamine at the time of their arrest [29], and 25% of violent offenders are under the influence of drugs and alcohol when they commit crimes [30]. The CJS bears a disproportionate burden of the epidemic of addiction, with up to 65% of prison inmates meeting DSM-IV criteria for drug or alcohol abuse or addiction [28, 30, 31]. In jails, in which inmates are either unsentenced or sentenced to shorter terms, women report even higher rates of substance use disorders (SUDs) than their male counterparts [32]. Up to 70% of HIV-infected prisoners meet criteria for opioid dependence [33]. These ongoing and often untreated SUDs play a major role in prison recidivism and likely contribute to poor HIV outcomes after release.

#### 3.1.2. Areas for Future Empirical Research

SUDs have a seemingly profound impact on the health outcomes of HIV-infected prisoners, for whom addiction-related lapses in HAART continuity threaten virologic suppression after release. With some notable exceptions described here, there have been relatively few published studies regarding the effect of SUDs on HIV outcomes in released prisoners. A 2008 study of HIV-infected reincarcerated jail detainees examined HAART adherence between the time of prison release and reincarceration. Findings revealed that those subjects who discontinued HAART were three times more likely to have also relapsed to marijuana or injection drug use between incarcerations [34]. Among HIV-infected injection drug users in Vancouver, any alcohol use or incarceration in the prior six months was associated with poor HAART adherence and worse virologic outcomes [35]. In a smaller study of 30 HIV-infected recently released prisoners, only 18 (60%) were enrolled in HIV primary care 21 days after incarceration. Enrollment in primary care was associated with abstinence from alcohol though this association did not reach statistical significance, likely owing to the small sample size [36]. Other published research on reduced uptake in HIV care after prison release has not assessed the important contribution of SUDs to discontinuous care [22, 23].

Extrapolating from studies of community-based subjects with HIV outside of the CJS, alcohol use is associated with decreased initiation of and adherence to HAART as well as increased rates of virologic failure [37]. Similarly, relapse to drug use, especially injection drugs and crack cocaine, has been associated with decreased HAART adherence, decreased engagement in HIV care, and increased risk of developing AIDS-defining conditions [5, 38]. The field would benefit from parallel research on the effect of SUDs on HIV biologic outcomes among prisoners and releasees living with HIV.

#### 3.1.3. Areas for Future Interventional Research

Despite overwhelming demonstrated need, substance abuse treatment programs during incarceration are relatively nonexistent [39]. As few as 11% of inmates with SUDs receive treatment for addiction during their incarceration with an emphasis instead on education, detoxification, and abstinence [28, 40]. Perhaps as a result, released prisoners are at extremely high risk for relapse to drugs or alcohol [41–43] with associated drug-related recidivism to prison [44] and excess mortality due to drug overdose [45, 46].

 Treatment of SUDs is clearly beneficial for relapse prevention but may also improve engagement in HIV care. Indeed, two recent interventions [33, 47] have demonstrated that opioid agonist therapies initiated upon release are effective at preventing relapse to opioid use and secondarily maintain HAART-related benefits achieved during incarceration. Since persistent virologic suppression is associated with decreased risk of HIV transmission, treatment of SUDs might, indirectly, be an effective secondary prevention measure for HIV [48, 49]. Treatment of SUDs is necessary but may be insufficient alone because of the many other complex social and behavioral stressors that simultaneously disrupt care in this patient population [28, 50]. These will be addressed systematically in the following sections.

### 3.2. Comorbid Mental Illness

#### 3.2.1. Epidemiology of the Problem

Substance use disorders are often concurrent and synergistic with mental health disorders and it may be difficult to disentangle the two issues. Over three-quarters of jail inmates with mental health disorders meet criteria for substance dependence or abuse [51]. Mental illness is highly prevalent in the CJS overall with 14–24% of prison and jail inmates reporting recent mental health problems [51]. Prevalence of psychiatric disorders is even higher among female compared to male inmates [32, 51]. The convergence of mental health disorders and HIV is especially evident in the CJS. Among inmates in the Texas Department of Criminal Justice, psychiatric disorders were more common among PLWHA compared to their un-infected peers [52].

#### 3.2.2. Areas for Future Empirical Research

Little is known about the effects of mental health disorders on HIV outcomes in dually diagnosed persons in the CJS. In non-CJS settings, however, treatment of psychiatric comorbidities has a profound impact on adherence to antiretroviral therapy. Several studies have examined the association of depression with HAART nonadherence in community-based cohorts. Among HIV-infected women, depression has been associated with decreased virologic response to HAART, higher likelihood of immunologic failure, and higher risk for all-cause mortality [53]. The longitudinal Multicenter AIDS Cohort Study of men who have sex with men showed that depression increased risk of interrupting or discontinuing HAART [54]. 

Furthermore, relatively few studies have examined associations between serious mental illness (SMI) and HAART adherence despite significant epidemiologic overlap between HIV and SMI. SMI includes the diagnoses of schizophrenia, schizoaffective disorder, bipolar depression, and major depression with psychotic features. Adherence to psychiatric medications among SMI patients ranges between 40–60%, suggesting that adherence to HAART in these populations might also be problematic [55]. This has especially troubling implications in the CJS where both HIV and SMI are highly concentrated. Approximately 14–22% of inmates in state and federal prisons/jails meet criteria for mania and 8–17% meet criteria for a psychotic disorder [51]. Interestingly, in one study of 47 community-based participants with SMI and HIV in Los Angeles, the mean adherence rate to antiretrovirals (proportion of prescribed doses taken) was 66% with surprisingly almost half of participants having a >90% adherence rate to HAART. The authors note that this signifies that HAART adherence rates are similar to adherence rates among people living with HIV but without SMI in community clinics, implying that diagnosis of comorbid SMI should not preclude HIV treatment [55]. This, however, was only one small study of community-based patients with comorbid SMI and HIV. To our knowledge, there are no published studies examining antiretroviral or psychiatric medication adherence among subjects with both SMI and HIV in the CJS. Given the large overlap between HIV and mental health disorders in this setting, more research is needed on correlates of HAART adherence in the correctional population with dual diagnoses.

#### 3.2.3. Areas for Future Interventional Research

Management of mental illness in correctional settings is an understudied but critical issue for people living with HIV. Among depressed HIV-infected patients in a non-correctional managed care setting, those who were treated with selective serotonin reuptake inhibitors for depression were more likely to be >95% adherent to HAART medications than those with untreated depression [56]. In addition, depressed patients adherent to psychiatric medications had similar HAART adherence and HAART-related outcomes as non-depressed people living with HIV on HAART [56]. Another study of injection drug users enrolled in a trial of directly administered antiretroviral therapy found that improvements in depressive symptoms were associated with increased HAART adherence and increases in CD4 cell counts [57]. Treatment of depression and serious mental illness can lead to improved HIV outcomes and HAART adherence rates that reach parity with people living with HIV but without mental illness [55, 56]. 

Unfortunately, even if inmates are engaged in psychiatric care during incarceration, treatment of mental illness in the CJS is often disrupted after release to communities. Upon initial release, suicide is common among former inmates, ranking among the top 5 causes of death in one study of released prisoners in Washington state and indicating inadequate treatment of mental health disorders during this critical transition period [45]. System-based structural barriers to care may be seemingly insurmountable. Understaffing of outpatient psychiatric clinics and insurer-based restrictions on mental health coverage can lead to the unavailability of treatment for mentally ill persons in the community. This is of grave concern for mentally ill patients with HIV because, as aforementioned, undertreated mental illness is related to decreased HAART adherence, worse biological outcomes, and increased risk of death [45, 53, 54, 57]. In addition, psychiatric disorders are highly associated with prison recidivism among both HIV-infected and uninfected individuals [21, 51, 58]. 

Methods to coordinate psychiatric services upon release from correctional settings are necessary. Formal case management services that enhance linkages to psychiatric care may secondarily improve adherence to HAART, and research is currently ongoing in this field [59]. Other strategies of diverting mentally ill offenders to treatment rather than incarceration through the use of mental health courts have been shown successful in engaging individuals in psychiatric care and cost savings compared to incarceration. The impact of these programs on HIV outcomes is not known but would be an important area of interest in future interventional studies [58, 60].

### 3.3. Homelessness

#### 3.3.1. Epidemiology of the Problem

A relative lack of mental health services in the community combined with the “War on Drugs” has resulted in the shuttling of mentally ill persons between homelessness and incarceration. Incarceration increases the risk of homelessness through loss of employment, loss of housing, and disruption of social support or community resources [61, 62]. Conversely, homelessness increases incarceration risk through common behaviors, including substance use and transactional sex [61–65]. Homelessness itself is also frequently criminalized, such that public intoxication, loitering, and vagrancy are behaviors punishable by incarceration. Approximately 23–68% of homeless individuals have a history of incarceration [61, 63]. Prevalence of homelessness prior to incarceration is twice as high among those with mental health problems as those without (13.2% versus 6.3% in state prisons) [51]. As a result, a revolving door between homelessness and incarceration is common, especially among those with mental illness or substance abuse issues [58, 61, 66]. 

Among PLWHA, homelessness and unstable housing have been associated with poor HIV outcomes including worse adherence to HAART, fewer ambulatory care visits, and increased risk of death [67–70]. A case-control study of the impact of housing on survival of persons with AIDS noted that 9.8% of persons were homeless at time of AIDS diagnosis and that 5-year survival was far worse for those who were homeless compared to those who were housed (67% versus 81%, resp.) [69]. This study also noted that antiretroviral use was lower among the homeless compared to the housed and that provision of housing improved survival [69]. In Chicago, provision of housing to HIV-infected homeless subjects with recent hospitalizations improved AIDS-free survival at 12 months [67]. Similarly, a prospective three-city study (in Baltimore, Chicago, and Los Angeles) randomized homeless or unstably housed PLWHA to immediate enhanced rental assistance versus customary housing services. In an intention to treat analysis, there was no difference between the two groups in terms of adherence to HAART, HIV RNA levels, or CD4 lymphocyte counts. In an “as-treated” analysis; however, those who remained homeless at followup were significantly more likely to have a higher HIV RNA viral load, regardless of randomization group [70]. Homelessness or unstable housing can have a profound impact on HAART adherence because homeless individuals are often distracted by trying to meet basic needs of food and shelter. The homeless often have difficulty safely storing medications although few current first-line antiretroviral regimens require refrigeration. Nevertheless, HAART may be successfully initiated in homeless or marginally housed patients: the three-city study did find that 78% of the homeless or unstably housed self-reported 100% past 2-day adherence to HAART [71]. 

The effects of homelessness on HAART adherence are often confounded by the presence of comorbid mental illness or SUDs. Homeless or unstably housed individuals are less likely to be adherent to HAART if they have recent substance use or higher depression scores [71]. A randomized controlled trial of HIV-infected homeless or marginally housed adults with depression found that providing antidepressants increased probability of achieving HIV virologic suppression [72]. Thus, treatment of HIV among the homeless or marginally housed can be complicated by comorbid substance use or mental health issues but proactive treatment of these comorbid conditions can improve HIV outcomes.

#### 3.3.2. Areas for Future Empirical Research

Although these studies have demonstrated the negative impact of homelessness on HAART adherence, there is no scientific literature concerning the impact of homelessness on recently incarcerated, HIV-infected populations. Given the high prevalence of homelessness after release from prison and the demonstrated beneficial effects of housing on social, psychiatric, addiction and HIV-related outcomes, future research should focus on correlates of risk of homelessness among recently-released prisoners and jail detainees.

Obtaining housing constitutes a top priority often expressed by HIV-infected released prisoners. Many prisoners return to communities plagued by poverty, unemployment, and violence—the same communities from which they were incarcerated. They must attempt to navigate this environment while simultaneously managing the more personal stresses of reuniting with parents, partners, and children after incarceration. Ideally, these relationships would support positive health promotion. All too often, however, these relationships are afflicted with addiction and interpersonal violence that only exacerbate stress and interfere with health-seeking behaviors. Returning prisoners must also attempt to manage comorbid medical illnesses, like diabetes and hypertension, which can become immediately life-threatening when undertreated, especially in the context of homelessness. These overlapping stresses (poverty, neighborhood characteristics, interpersonal relationships, and comorbid chronic medical conditions) may be important to individuals' health after prison release and likely contribute to prison recidivism. Unfortunately, there have been few published studies to date around homelessness and HIV-related continuity of care after release, thus limiting our discussion on these topics. Future studies should address the impact of homelessness and social instability, as more broadly defined, on healthcare utilization patterns of PLWHA after release from prison.

#### 3.3.3. Areas for Future Interventional Research

Despite the strong association between homelessness and incarceration, strategies addressing the housing needs of HIV-infected inmates upon release have not been well studied. A possible strategy that may improve comorbid substance use or psychiatric disorders as well as recidivism is a Housing First approach. Contrary to traditional substance abuse or mental health treatment programs which require strict adherence to treatment in order to maintain housing, Housing First programs provide immediate access to permanent housing without any prerequisites for psychiatric treatment or sobriety. Among homeless or marginally housed persons with serious mental illness, Housing First programs have been successful in improving residential status and decreasing use of inpatient, emergency, and criminal justice system services [73, 74]. Although Housing First models have not been studied in the HIV-infected recently incarcerated population, its successes among vulnerable populations with serious mental illness or substance use disorders suggest it may be a promising strategy for this group. 

Other possible interventions to improve HIV outcomes among homeless or marginally housed releasees involve improving engagement to care through increased supportive services. In San Francisco, a clinic specializing in supporting recently released inmates in the transition back to the community was noted to successfully engage a population in which 38% were homeless [75]. The clinic employed a full-time community health worker who attended parole meetings and provide enhanced case management services for clinic patients. Although the San Francisco clinic targeted non-HIV-infected persons, the transitions clinic may serve as a model for maintaining HIV-infected, homeless individuals in care. Currently, the HRSA-funded Enhancing Linkages to HIV Primary Care and Services in Jail Settings Initiative is studying the impact of enhanced case management services that can include housing services [59]. Although enhanced case management services may not improve linkage to care in all settings, [76] its efficacy among homeless populations has not yet been studied. 

 Fortunately, homelessness is a modifiable risk factor that could be prevented with effective discharge planning services. Transitional case management programs should proactively address housing instability as part of a comprehensive package of social services, recognizing the extreme vulnerability during this period of movement between prisons and communities. Provision of housing is, like many of the other issues addressed here, not a stand-alone problem.

### 3.4. Loss of Medical and Social Benefits

#### 3.4.1. Epidemiology of the Problem

In the current USA healthcare system, where medical care is predominantly funded by third-party payors, gaps in insurance coverage during incarceration contribute to fragmentation of care upon release. By virtue of their socioeconomic or medical disability status, most inmates with health insurance have Medicaid prior to incarceration. Medicaid prohibits any use of federal funds for medical or psychiatric care of inmates in jails and prisons. Most states (>90%) terminate Medicaid coverage during incarceration [77]. For those who qualify for Medicaid by virtue of their receiving social security income, termination, rather than suspension, of benefits is almost guaranteed [77, 78]. Termination signifies that inmates are completely removed from Medicaid rolls and are required to reapply. Because of the difficulties former inmates face in obtaining employment, employment-based health insurance is not a reliable option. In addition, job applications for even the most menial opportunities often require a permanent address, which many former inmates cannot provide [79]. During incarceration, many other social benefits including food stamps, state-subsidized housing, and temporary assistance for needy families are also suspended or discontinued [80]. 

As a result, many inmates are left with gaps in medical and social benefit coverage upon release from prison. Reapplication procedures for Medicaid can be initiated as early as 45–90 days prior to expected release [78] but require photo identification and other documentation that are sometimes logistically difficult for former inmates to obtain. Furthermore, released prisoners often are returned to communities late at night or on weekends when services are closed [79]. Because of these barriers, a survey of 511 women leaving New York's jails found that only half of the participants had obtained health insurance by one year after release, regardless of whether or not they had comorbid conditions like asthma, diabetes, or HIV that required ongoing medical care [81]. Interruptions in coverage result in a greater reliance on “free” episodic emergency and hospital-based care [82] and decreased engagement with mental health services for the mentally ill [83]. Perhaps because having medical insurance is a marker of social stability, subjects who obtained health insurance coverage following release were 69% less likely to be rearrested for any cause and 91% less likely to be rearrested on drug-related charges at the time of follow-up compared to those without health insurance [44].

For HIV-infected released prisoners, continuity of medical coverage is especially crucial for continued provision of HAART. In the past, coverage for antiretrovirals was dismal: in a 1994 study, 19% of HIV-infected prisoners reported trying to get rearrested just to obtain prison-based HIV care that was seemingly not available to them in the community after release [84]. Fortunately today, coverage gaps are bridged by the AIDS Drug Assistance Program (ADAP) which, under HRSA's Ryan White HIV/AIDS Treatment Modernization Act Part B, acts as the “payor of last resort.” [85] Baillargeon et al. [22] estimated that 100% of HIV-infected inmates who received HAART during incarceration in Texas would qualify for ADAP. Over the past decade, ADAP has made significant inroads into covering this vulnerable population.

#### 3.4.2. Areas for Future Empirical Research

Beyond the availability of medication assistance programs, there remains a significant knowledge gap about why and how released prisoners experience decreased persistence in HIV care. Certainly the problem is multifactorial and complex: this population is often marginalized from the mainstream medical community because of active drug use, mental illness, or HIV-related stigma prior to and following incarceration. Even in Canada, in which a nationalized healthcare system removes significant insurance-related barriers to care, released prisoners report problems obtaining appointments with an HIV clinician after return to the community [86]. These appointment delays have been associated with lapses in medication adherence lasting 1–3 days which, especially in the case of antiretrovirals with low genetic barriers to resistance, may be sufficient to result in virologic failure. Other lapses in HAART adherence have been specifically linked to points of custody transfers (i.e., movement from police stations or court to correctional facilities, transfer from prisons to communities.) [86]. Future research around correlates of decreased persistence in HIV care for released prisoners should incorporate mixed methods with qualitative interviews of released prisoners to increase granularity of available data.

#### 3.4.3. Areas for Future Interventional Research

In spite of significant safety nets for people living with HIV, there remain gaps in medical coverage after release that present barriers to continuous HIV care. These should be addressed by future interventions. Delays in completing and processing ADAP applications are a limiting factor in HIV-infected recently released prisoners benefiting from the program. In a cross-sectional mixed methods study of 105 HIV-infected recently released prisoners in Florida, 19% reported that the paperwork required to obtain medical care, insurance, and HAART presented a major obstacle to care [79]. Of 177 HIV-infected reincarcerated jail inmates, 48% had no health insurance and only 61% had ADAP when living in the community [34]. Of those who reported taking HAART in the community, 19% never once saw an HIV provider during the 12-month-observation period [34]. Conversely, inmates who received assistance with ADAP applications in Texas prior to release were three times more likely to fill HAART prescriptions within 10 days after release [22]. This latter study in Texas was one of the few to date that incorporated assistance with ADAP applications into a comprehensive transitional case management program. Future interventions should include similar programs while taking an evidence-based approach to measuring important outcomes, including HAART adherence, continuity of care, enrollment in an HIV clinic, prison recidivism, and HIV-associated morbidity.

## 4. Summary

Each year, almost 10 million people are released from USA jails and prisons to the community [3, 44]. A small but significant proportion of these released prisoners are living with HIV, a disease best managed with lifelong combination antiretroviral therapy in regular consultation with an HIV specialist. Continuity of care is critical for prevention of HIV-related morbidity and mortality but faces numerous obstacles in the transitions from community to prison and back to the community. In this paper we explored four major challenges to successful management of HIV in correctional populations: relapse to substance use, mental illness, homelessness, and loss of medical and social benefits. Each of these areas constitutes a competing priority upon release that demands immediate attention and diverts time, energy, and scarce resources away from engagement in care. In this era of HAART, HIV may be perceived as a chronic, asymptomatic disease of less urgent interest to returning prisoners than meeting basic subsistence needs, coping with debilitating psychiatric illnesses or meeting the physical and mental demands of powerful addictions. These barriers to continuity of HIV care and HAART adherence must be addressed for the sake of the individual and from a public health standpoint. 

The numerous outstanding knowledge gaps and solution gaps concerning HIV outcomes in criminal justice populations also present exciting academic opportunities for future research. We have attempted to shed light on these gaps in support of a contemporary research agenda. Evidence-based examinations of the problems and solutions facing criminal justice populations are critical to progress in the management of HIV in correctional systems.

## 5. Discussion

Our review of the literature suggests that linkages from prison-based to community HIV care are critical for maintaining the benefits of HAART achieved during incarceration. There is ongoing debate in the criminal justice field about how best to design transitional interventions to address these complex issues that otherwise disrupt care for returning prisoners. Options proposed thus far include intensive case management, supportive housing, opioid substitution therapy, directly administered antiretroviral therapy, medication adherence support, or combinations therein. We advocate that, regardless of methodology, the most effective interventions require significant breadth to address all of the barriers that manifest during transfer from prison to the community. 

Unfortunately, there are significant logistical constraints to introducing evidence-based interventions into correctional systems either during incarceration or prior to release. Inmates are often transitory, cycling frequently and rapidly between jails and communities or among various correctional facilities. This migratory pattern provides a narrow window in which to introduce and assess any given intervention. Constraints may also be imposed by a correctional system itself that enforces strict privacy protection measures (especially regarding HIV serostatus) and is subject to state, or federally based budgetary limitations. Even outside prison walls, and thus, technically outside of correctional systems, released prisoners may be difficult subjects in longitudinal studies because they often experience high rates of attrition. Strategies for collaboration between academic centers of research and correctional systems have been recently proposed elsewhere [87].

Despite these challenges, empirical and interventional research is strongly needed to improve the management of HIV both during and following incarceration. Future research must work to develop geographically and culturally appropriate interventions that are highly tailored to individual needs. Perhaps most difficult, future research should attempt to deconstruct structural barriers to persistence in HIV care that include termination of medical/social benefits during incarceration, minimal linkages to community-based psychiatric and HIV primary care, and often-absent treatment for substance use disorders. As providers and clinical researchers, we must act as advocates for this often silenced and marginalized population living with HIV.
